# Supplementary material for: E3 ubiquitin ligase Bre1 couples sister chromatid cohesion establishment to DNA replication in Saccharomyces cerevisiae
Source: eLife. 2017 Oct 23;6:e28231. doi: 10.7554/eLife.28231 (PMC5699866; doi:10.7554/eLife.28231)
Supplement: Supplementary file 1. [file elife-28231-supp1.docx]

**Supplementary File 1**

**Yeast strains used in this study**

| Strain name | Genotype | Reference |
| --- | --- | --- |
| YPH768 | *MAT a met15-∆0 ura3-52 lys2-801(amber) ade2-101(ochre) trp1-∆1 leu2-∆1 URA3 SUP11 CFIII (CEN3.L)* | Philip Hieter lab |
| YPH1343 (AFS479) | *MAT a met15-∆0 ade2-1 can1-100 ura3-1 trp1-1 leu2-3,112::lacO-256(pAFS59)-LEU2^+^*  *his3-11,15::GFP13-LacI(pAFS144)::HIS3^+^* | (Straight et al., 1996) |
| YPH499 | *MAT a met15-∆0 ura3-52 lys2-801 ade2-101 his3-∆200 trp1-∆63 leu2-∆1* | (Sikorski and Hieter, 1989) |
| WYYY34 | *YPH768; bre1Δ::TRP1* | This study |
| WYYY120 | *YPH768; lge1Δ::TRP1* | This study |
| WYYY42 | *YPH768; ctf18Δ::TRP1* | This study |
| WYYY187 | *YPH768; ctf4Δ::KanMX4* | This study |
| WYYY186 | *YPH768; ctf18Δ::KanMX4 bre1Δ::TRP1* | This study |
| WYYY184 | *YPH768; ctf18Δ::KanMX4 lge1Δ::TRP1* | This study |
| WYYY189 | *YPH768; ctf4Δ::KanMX4 lge1Δ::TRP1* | This study |
| WYYY192 | *YPH768; ctf4Δ::KanMX4 bre1Δ::TRP1* | This study |
| WYYY204 | *YPH1343; Flag-HTB1-K123R::URA3* | This study |
| WYYY215 | *YPH1343; Flag-HTB1::URA3* | This study |
| WYYY246 | *YPH1343; htb2Δ::KanMX6 Flag-HTB1::URA3* | This study |
| WYYY232 | *YPH768; SCC1-3HA::KanMX6* | This study |
| WYYY236 | *YPH768; SCC1-3HA::KanMX6 bre1Δ::TRP1* | This study |
| WYYY238 | *YPH768; SMC3-3HA::KanMX6* | This study |
| WYYY242 | *YPH768; SMC3-3HA::KanMX6 bre1Δ::TRP1* | This study |
| WYYY250 | *YPH1343; Bre1-AID*-9Myc::KanMX4* | This study |
| WYYY326 | *YPH1343; Bre1-AID*-9Myc::KanMX4 URA3:: ADH1-OsTIR1-9myc* | This study |
| WYYY108 | *YPH1343; bre1Δ::KanMX6* | This study |
| WYYY21 | *YPH1343; lge1Δ::KanMX6* | This study |
| WYYY130 | *YPH1343; bre1Δ::KanMX6 lge1Δ::TRP1* | This study |
| WYYY19 | *YPH1343; ctf18Δ::KanMX6* | This study |
| WYYY140 | *YPH1343; ctf18Δ::KanMX6 bre1Δ::TRP1* | This study |
| WYYY132 | *YPH1343; ctf18Δ::KanMX6 lge1Δ::TRP1* | This study |
| WYYY223 | *YPH1343; ctf4Δ::KanMX6* | This study |
| WYYY138 | *YPH1343; ctf4Δ::KanMX6 bre1Δ::TRP1* | This study |
| WYYY112 | *YPH1343; ctf4Δ::KanMX6 lge1Δ::TRP1* | This study |
| WYYY248 | *YPH1343; Bre1-RINGΔ::KanMX6* | This study |
| WYYY215 | *YPH1343; FLAG-HTB1::URA3* | This study |
| WYYY246 | *YPH1343; htb2Δ::KanMX6 FLAG-htb1-K123R::URA3* | This study |
| WYYY323 | *YPH1343; URA3:: ADH1-OsTIR1-9myc* | This study |
| WYYY278 | *YPH499; Ctf18-3HA::KanMX6* | This study |
| WYYY294 | *YPH499; Ctf18-3HA::KanMX6 bre1Δ::TRP1* | This study |
| WYYY276 | *YPH499; Ctf4-3HA::KanMX6* | This study |
| WYYY336 | *YPH499; Ctf4-3HA::KanMX6 bre1Δ::TRP1* | This study |
| WYYY280 | *YPH499; Eco1-3HA::KanMX6* | This study |
| WYYY304 | *YPH499; Eco1-3HA::KanMX6 bre1Δ::TRP1* | This study |
| WYYY284 | *YPH499; Pol1-13Myc::KanMX6* | This study |
| WYYY302 | *YPH499; Pol1-13Myc::KanMX6 bre1Δ::TRP1* | This study |
| WYYY636 | *YPH499; Psf2-13Myc::KanMX6* | This study |
| WYYY638 | *YPH499; Psf2-13Myc::KanMX6 bre1Δ::TRP1* | This study |
| WYYY640 | *YPH499; Mcm10-13Myc::KanMX6* | This study |
| WYYY642 | *YPH499; Mcm10-13Myc::KanMX6 bre1Δ::TRP1* | This study |
| WYYY718 | *YPH499; Ctf4-13Myc::KanMX6* | This study |
| WYYY726 | *YPH499; Eco1-13Myc::KanMX6* | This study |
| WYYY728 | *YPH499; Eco1-13Myc::KanMX6 bre1Δ::TRP1* | This study |
| WYYY767 | *YPH1343; htb2∆::KanMX6* | This study |
| WYYY769 | *YPH1343; swd2∆::KanMX6* | This study |
| WYYY792 | *YPH499; Ctf4-13Myc::KanMX6 bre1-RING∆::TRP1* | This study |
| WYYY794 | *YPH499; Mcm10-13Myc::KanMX6 bre1-RING∆::TRP1* | This study |
| WYYY798 | *YPH499; CTF4-13Myc::KanMX6 htb1-K123R::URA3 htb2∆::TRP1* | This study |
| WYYY802 | *YPH499; Mcm10-13Myc::KanMX6 htb1-K123R::URA3 htb2∆::TRP1* | This study |
| WYYY859 | *YPH499; Ctf4-13Myc::KanMX6 Mcm10-3HA::TRP1* | This study |
| WYYY860 | *YPH499; Ctf4-13Myc::KanMX6 Mcm10-3HA::TRP1 bre1∆::LEU2* | This study |
| WYYY861 | *Mcm10-13Myc::KanMX6 (CEN URA3 FLAG-Bre1)* | This study |
| WYYY863 | *YPH499; Smc3-13Myc::KanMX6 (CEN URA3 FLAG-Bre1)* | This study |
| WYYY865 | *YPH499 (CEN URA3 FLAG-Bre1)* | This study |
| WYYY879 | *YPH499; Smc3-13Myc::KanMX6* | This study |
| WYYY881 | *YPH499; Smc3-13Myc::KanMX6 bre1Δ::TRP1* | This study |
| WYYY910 | *YPH1343; rad6Δ::KanMX6* | This study |
| WYYY940 | *YPH1343; Bre1-3HA::TRP1* | This study |
| WYYY942 | *YPH1343; RINGΔ-3HA::TRP1* | This study |
| WYYY944 | *YPH1343;* *htb2Δ::KanMX6 FLAG-htb1-K123R::URA3 Bre1-3HA::TRP1* | This study |

**Reference**

Sikorski, R.S., and Hieter, P. (1989). A system of shuttle vectors and yeast host strains designed for efficient manipulation of DNA in Saccharomyces cerevisiae. Genetics *122*, 19-27.

Straight, A.F., Belmont, A.S., Robinett, C.C., and Murray, A.W. (1996). GFP tagging of budding yeast chromosomes reveals that protein–protein interactions can mediate sister chromatid cohesion. Current Biology *6*, 1599-1608.
