# Supplementary material for: E3 ubiquitin ligase Bre1 couples sister chromatid cohesion establishment to DNA replication in Saccharomyces cerevisiae
Source: eLife. 2017 Oct 23;6:e28231. doi: 10.7554/eLife.28231 (PMC5699866; doi:10.7554/eLife.28231)
Supplement: Supplementary file 2. [file elife-28231-supp2.docx]

**Supplementary File 2**

**Yeast plasmids used in this study**

| Plasmid name | Description | Source |
| --- | --- | --- |
| WYYp19 (pRS316-Flag-*HTB1*) | (Flag-*HTB1* in pRS306):CEN *URA3* | (Robzyk et al., 2000) |
| WYYp21 (pRS413-Flag-*HTB1*K123R) | (Flag-*HTB1K123R* in pRS413):CEN *HIS3* | (Robzyk et al., 2000) |
| WYYp30 (pRS316- -Flag-*HTB1*K123R) | (Flag-*HTB1*K123R in pRS316): CEN *URA3* | This study |
| WYYp74 (pKan-AID*-9Myc) | Epitope tagged-AID* cassette in pSM409 with KanMX4 selection marker | (Morawska and Ulrich, 2013) |
| WYYp64 (pNHK53) | Integration plasmid containing OsTIR1-9Myc cassette and *URA3* | (Nishimura et al., 2009) |
| WYYp179 | FLAG-Bre1 p416 ADH: CEN URA3 pADH1-FLAG-BRE1 | (Wozniak and Strahl, 2014) |

**Reference**

Morawska, M., and Ulrich, H.D. (2013). An expanded tool kit for the auxin‐inducible degron system in budding yeast. Yeast *30*, 341-351.

Nishimura, K., Fukagawa, T., Takisawa, H., Kakimoto, T., and Kanemaki, M. (2009). An auxin-based degron system for the rapid depletion of proteins in nonplant cells. Nature methods *6*, 917-922.

Robzyk, K., Recht, J., and Osley, M.A. (2000). Rad6-dependent ubiquitination of histone H2B in yeast. Science *287*, 501-504.

Wozniak, G.G., and Strahl, B.D. (2014). Catalysis-dependent stabilization of Bre1 fine-tunes histone H2B ubiquitylation to regulate gene transcription. Genes & development *28*, 1647-1652.
