# Supplementary material for: E3 ubiquitin ligase Bre1 couples sister chromatid cohesion establishment to DNA replication in Saccharomyces cerevisiae
Source: eLife. 2017 Oct 23;6:e28231. doi: 10.7554/eLife.28231 (PMC5699866; doi:10.7554/eLife.28231)
Supplement: Supplementary file 3. [file elife-28231-supp3.docx]

**Supplementary File 3**

**Sequences of primers used in RT-qPCR experiments in this study**

| Primer name | Primer sequence | Purpose | Source |
| --- | --- | --- | --- |
| CLN2-F | TATCCCAGGATAGTGATGCCACTG | Test *CLN2* mRNA levels | (Zimmermann et al., 2011) |
| CLN2-R | TCTAAGTAAGTCGTACTGCCACGC | Test *CLN2* mRNA levels | (Zimmermann et al., 2011) |
| CLB5-F | GCCCAACCCACTCAATTTCCTAAG | Test *CLB5* mRNA levels | (Zimmermann et al., 2011) |
| CLB5-R | GAATGAATTGGTGGCAGCAGTAGG | Test *CLB5* mRNA levels | (Zimmermann et al., 2011) |
| ACT1-F | ACGTGAGTAACACCATCACCGGAA | Test *ACT1* mRNA levels | (Zimmermann et al., 2011) |
| ACT1-R | TCCAGCCTTCTACGTTTCCATCCA | Test *ACT1* mRNA levels | (Zimmermann et al., 2011) |
| SCC1-F | CTTGGTGGACCCAATAAGCTG | Test *SCC1* mRNA levels | This study |
| SCC1-R | AATACTACCCATGCACCAGCG | Test *SCC1* mRNA levels | This study |
| SMC3-F | TTAAAGAAGTTGCCCGCTTGC | Test *SMC3* mRNA levels | This study |
| SMC3-R | GAAGCCAAGTGGTGGATTTGC | Test *SMC3* mRNA levels | This study |
| CTF4-F | ATACATGTCTGGCCCTTGGC | Test *CTF4* mRNA levels | This study |
| CTF4-R | CTCCATTTCGGATGGCAACG | Test *CTF4* mRNA levels | This study |
| CTF18-F | TGGCTCAAGGTGACGTAAGG | Test *CTF18* mRNA levels | This study |
| CTF18-R | GGGGGAGTCCTTGTTGGATG | Test *CTF18* mRNA levels | This study |
| ECO1-F | TAGTCGTGGACGTTGGATGG | Test *ECO1* mRNA levels | This study |
| ECO1-R | ACCCAACTTCCTTGCTGTCC | Test *ECO1* mRNA levels | This study |

**Reference**

Zimmermann, C., Chymkowitch, P., Eldholm, V., Putnam, C.D., Lindvall, J.M., Omerzu, M., Bjørås, M., Kolodner, R.D., and Enserink, J.M. (2011). A chemical-genetic screen to unravel the genetic network of CDC28/CDK1 links ubiquitin and Rad6–Bre1 to cell cycle progression. Proceedings of the National Academy of Sciences *108*, 18748-18753.
